# Heritability of the Human Infectious Reservoir of Malaria Parasites

**DOI:** 10.1371/journal.pone.0011358

**Published:** 2010-06-29

**Authors:** Yaye Ramatoulaye Lawaly, Anavaj Sakuntabhai, Laurence Marrama, Lassana Konate, Waraphon Phimpraphi, Cheikh Sokhna, Adama Tall, Fatoumata Diène Sarr, Chayanon Peerapittayamongkol, Chalisa Louicharoen, Bradley S. Schneider, Anaïs Levescot, Arthur Talman, Isabelle Casademont, Didier Menard, Jean-François Trape, Christophe Rogier, Jaranit Kaewkunwal, Thanyachai Sura, Issarang Nuchprayoon, Frederic Ariey, Laurence Baril, Pratap Singhasivanon, Odile Mercereau-Puijalon, Rick Paul

**Affiliations:** 1 Institut Pasteur de Dakar, Laboratoire d'Entomologie Médicale, Dakar, Senegal; 2 Institut Pasteur, Laboratoire de la Génétique de la réponse aux infections chez l'homme, Paris, France; 3 Institut Pasteur, Unité de Pathogénie Virale, Paris, France; 4 Institut Pasteur de Dakar, Unité d'Epidémiologie, Dakar, Senegal; 5 Faculté des Sciences et Techniques, UCAD, Dakar, Senegal; 6 Department of Tropical Hygiene, Faculty of Tropical Medicine, Mahidol University, Bangkok Thailand; 7 Institut de Recherche pour le Développement, Laboratoire de Paludologie, Dakar, Senegal; 8 Inter-Department Program of Biomedical Science, Faculty of Graduate School, Chulalongkorn University, Bangkok, Thailand; 9 Unité d'Epidémiologie Moléculaire, Institut Pasteur, Phnom Penh, Cambodia; 10 Institut de Médecine Tropicale du Service de Santé des Armées, Unité de Recherche en Biologie et épidémiologie parasitaires, IFR48, Le Pharo, Marseille, France; 11 Department of Medicine, Faculty of Medicine Ramathibodi Hospital, Mahidol University, Bangkok, Thailand; 12 Department of Pediatrics, Faculty of Medicine, Chulalongkorn University, Bangkok, Thailand; 13 Institut Pasteur, Unité d'Immunologie Moléculaire des Parasites, CNRS URA 2581, Paris, France; London School of Hygiene and Tropical Medicine, United Kingdom

## Abstract

**Background:**

Studies on human genetic factors associated with malaria have hitherto concentrated on their role in susceptibility to and protection from disease. In contrast, virtually no attention has been paid to the role of human genetics in eliciting the production of parasite transmission stages, the gametocytes, and thus enhancing the spread of disease.

**Methods and Findings:**

We analysed four longitudinal family-based cohort studies from Senegal and Thailand followed for 2–8 years and evaluated the relative impact of the human genetic and non-genetic factors on gametocyte production in infections of *Plasmodium falciparum* or *P. vivax*. Prevalence and density of gametocyte carriage were evaluated in asymptomatic and symptomatic infections by examination of Giemsa-stained blood smears and/or RT-PCR (for falciparum in one site). A significant human genetic contribution was found to be associated with gametocyte prevalence in asymptomatic *P. falciparum* infections. By contrast, there was no heritability associated with the production of gametocytes for *P. falciparum* or *P. vivax* symptomatic infections. Sickle cell mutation, HbS, was associated with increased gametocyte prevalence but its contribution was small.

**Conclusions:**

The existence of a significant human genetic contribution to gametocyte prevalence in asymptomatic infections suggests that candidate gene and genome wide association approaches may be usefully applied to explore the underlying human genetics. Prospective epidemiological studies will provide an opportunity to generate novel and perhaps more epidemiologically pertinent gametocyte data with which similar analyses can be performed and the role of human genetics in parasite transmission ascertained.

## Introduction

Transmission of malaria parasites from man to mosquito depends on the production of gametocyte sexual parasite stages in the human host that are subsequently taken up by a mosquito during a bloodmeal. For *Plasmodium falciparum*, the etiological agent of malignant tertian malaria, sexual stage differentiation (gametocytogenesis) from asexual parasites occurs in the blood of the human host. Both *in vitro* and *in vivo* studies emphasise the importance of environmental stimuli in modulating gametocytogenesis [1], [2]. Gametocyte production may occur in response to environmental factors that directly suppress asexual proliferation *in vitro* [3], but this has not been shown *in vivo* [4]. Gametocyte carriage has been associated with a worsening blood environment for the parasite (e.g. fever responses, anaemia, and the presence of reticulocytes) [5]–[7]. However, such cues are associated with symptomatic episodes of malaria and it is now well established that asymptomatic infections can also generate gametocytes and infect mosquitoes [8]–[10]. Molecular techniques have revealed extensive occurrence of sub-microscopic gametocytes [11], [12] that can infect mosquitoes [13] and play an important role as a reservoir of infection especially in areas of seasonal transmission [14]. No specific risk factors have yet been identified for gametocyte carriage in asymptomatic infections, although as in symptomatic infections, drug treatment of asymptomatic infections with sulfadoxine-pyrimethamine reduces gametocyte carriage [15].

Studies on human genetic factors associated with malaria have hitherto concentrated on their role in susceptibility to and protection from disease [16]. The most well-known is the sickle cell mutation (HbS) in Africa, which confers protection against severe malaria in heterozygotes, but causes fatal sickle cell disease in homozygotes [17], [18], illustrating the powerful selective pressure exerted by malaria on the human genome [19]. In contrast, virtually no attention has been paid to the role of human genetics in eliciting the production of gametocytes and thus enhancing the spread of parasites and hence disease. Differences in the tendency of sympatric ethnic groups to carry gametocytes have, however, been noted since 1914 [20] and more recently in Irian Jaya [21] and Burkina Faso [22]. The extent to which such differences are a consequence of the impact of host genetics on parasite asexual proliferation rather than directly on gametocytogenesis has not been addressed. Asexual parasite density has been repeatedly shown to be influenced by host genetics and the chromosomal region 5q31–33, which contains a cluster of cytokine genes, has been identified as important in the control of asexual parasite density [23]–[27]. Thus, the impact of human genetics on gametocyte production may occur via its effect on asexual parasite proliferation. The observed association of gametocytes with anaemia and subsequent erythropoietic response [5]–[7], [28]–[29] has yet to be explored genetically, despite a high prevalence of inherited blood disorders that induce anaemia, such as HbS and alpha-thalassaemia, in regions endemic for malaria [30]. Very recently, however, increased gametocyte carriage was observed in individuals with HbC [31].

Transmission success is crucial to the parasite and gametocyte production has been repeatedly shown to be under strong selective pressure [32], thus making this stage of the lifecycle propitious for intervention. Current efforts concentrate on the development of transmission-blocking vaccines [33] and exploration of parasite genes implicated in sexual development [34]. The possibility of using genome-wide association studies in humans potentially enables identification of critical molecular pathways in humans that influence gametocyte production, thereby potentially generating novel strategies for treatment and prevention; for example by using drug treatment targeting individuals genetically susceptible to carry gametocytes or developing novel drugs that target the human factors that induce gametocyte production. As a first step, however, it is necessary to establish the extent to which humans exert an influence on gametocyte production, in addition to the known intrinsic parasite clone variability in gametocyte production [35]. Measurement of heritability is central to quantitative genetic analysis and provides an estimate of the genetic basis underlying a trait.

In this study we examine the overall contribution of human host genetic factors (i.e. the heritability) to variation in gametocyte production in three longitudinal cohort studies occurring in areas of differing transmission intensity in Senegal, where *P. falciparum* is endemic, and in one cohort in Thailand where both *P. falciparum* and *P. vivax* are present. Moreover, we specifically examine the impact on gametocyte production of two inherited blood disorders known to cause anaemia, sickle cell trait [17] and alpha-thalassaemia [18], [36].

## Materials and Methods

### Ethics statement

#### Dielmo and Ndiop, Senegal

The project protocol and objectives were carefully explained to the assembled village population and informed consent was individually obtained from all subjects either by signature or by thumbprint on a voluntary consent form written in both French and in Wolof, the local language. Consent was obtained in the presence of the school director, an independent witness. For very young children, parents or designated tutors signed on their behalf. The protocol was approved by the Ethical Committee of the Pasteur Institute of Dakar and the Ministry of Health of Senegal (ethics S1). An agreement between Institut Pasteur de Dakar, Institut de Recherche pour le Développement (IRD) and the Ministère de la Santé et de la Prévention of Senegal defines all research activities in Dielmo and Ndiop villages. Each year, the project was re-examined by the Conseil de Perfectionnement de l'Institut Pasteur de Dakar and the assembled village population; informed consent was individually renewed from all subjects.

#### Gouye Kouly, Senegal

The project protocol and objectives were carefully explained to the assembled village population and informed consent was individually obtained from all subjects by signature on a voluntary consent form written in both French and in Wolof. The protocol was approved by the Ethical Committee of the Ministry of Health of Senegal (ethics S2).

#### Suan Phung, Thailand

The project protocol and objectives were explained to the population and signed informed consent was individually obtained from all study participants or their parents. Ethical permission for the study was granted by the Ethical Committee of the Ministry of Public Health of Thailand (ethics S3).

### Study sites and subjects

#### Dielmo and Ndiop, Senegal

The Dielmo and Ndiop longitudinal surveys have been described in detail elsewhere [37]–[39]. Briefly, a longitudinal cohort study of malaria has been carried out since 1990 in Dielmo and 1993 in Ndiop. For this analysis we use data acquired from 1990–1998 in Dielmo and 1993–1998 in Ndiop. In Dielmo there were 594 individuals from 190 nuclear families and in Ndiop 653 from 208 nuclear families. In each village, the majority of individuals were related to each other, forming one large complex family: 1 family of 453 individuals in Dielmo and one family of 503 in Ndiop. Overall there were 10 completely independent families in Dielmo and 21 in Ndiop. In Dielmo, the ethnic groups consisted of 79% Serere (Niominka: 59% and Sine/Baol: 20%), 11% Mandinka and 10% miscellaneous, whereas in Ndiop, there were 76% Wolof, 19% Fulani and 5% miscellaneous.

#### Gouye Kouly, Senegal

A family-based longitudinal cohort study was performed from June 2004–November 2005 in a third site in Senegal, Gouye Kouly. Family structures were constructed by using a questionnaire, interviewing each individual or key representatives of the household to obtain both demographic information such as birth date, age, sex and genetic relationships between children, their parents, and sometimes their grandparents or non-relatives in the same household, and other households. The population was composed of 482 individuals that belong to 9 independent families, one of which is a large complex family of 423 individuals that form 173 nuclear families. The majority of individuals were Serere.

#### Suan Phung, Thailand

In Thailand, a community-based cohort study was carried out from June 1998 to May 2005 [40]. The study was conducted in a mountainous area of Suan Phung district, Ratchaburi province, Thailand. Suan Phung is a small district situated near the Thai-Myanmar border. Suan Phung has a total population of 5,368 living in 7 hamlets, of which 3,484 villagers of all ages participated in the study. This community is made up of a group of 4 closely related ethnic groups, the majority of which are Karen (85%), some Thai (14%) and the rest are Mon and Burmese (1%). The total pedigrees are comprised of 2,427 individuals, including absent or deceased relatives. There were 238 independent families containing 603 nuclear families; the majority are 2 generation-families with family size range from 3 to 958. The recruitment procedure has been previously detailed [40].

### Malaria epidemiology

Malaria transmission is perennial in Dielmo, where a river maintains larval breeding sites for the mosquitoes even in the dry season. The number of infective bites per person per year (Entomological Inoculation Rate, EIR) is of the order of 200 [41]. By contrast, malaria transmission is strictly seasonal in Ndiop and dependent upon the rainy season that occurs from July–September and the EIR is approximately 20 [42]. Transmission is similarly highly seasonal in Gouye Kouly with EIR measured at approximately 2 infectious bites per person per year (unpubl. data). Such differing transmission has marked consequences on the epidemiology of malaria in the villages. This is most evident in the higher *P. falciparum* prevalence rates of infection in Dielmo (80%) compared to the seasonal rates in Ndiop that change from 20% in the dry season to 70% in the rainy season [39], [43] and from 8% to 15% in Gouye Kouly (unpubl. data).

The epidemiology of malaria in the Thai site has been described elsewhere [40]. Briefly, the incidence of malaria is highly seasonal with annual peaks in May–June and decreased over the duration of the study from 141 per 1000 person-years in 1999 to 57 in 2004 for *P. falciparum* and from 79 to 28 for *P. vivax*. *P. falciparum* prevalence rates varied from 1–7% seasonally and from 1–4% for *P. vivax*. There was good concordance in the population prevalence of fevers that were found to be positive for malaria parasites and the fraction of fevers attributable to malaria. Thus, in this site, virtually all infections lead to febrile episodes. Peak incidence occurred in an earlier age group (5–9 years old) for *P. vivax* than for *P. falciparum* (10–15 years old). Parasite densities of either species peaked in the <10 years old age group.

### Data Collection

#### Symptomatic episodes (passive case detection)

The installation of health clinics in each of the study sites enabled passive case detection of malaria episodes. We defined clinical malaria episodes as measured fever (axillary temperature >37.5°C) or fever-related symptoms (headache, vomiting, subjective sensation of fever) associated with i) a *P. falciparum* parasite/leukocyte ratio higher than an age-dependent pyrogenic threshold previously identified in the patients from Dielmo [44], ii) a *P. falciparum* parasite/leukocyte ratio higher than 0.3 parasite/leukocyte in Ndiop, iii) a slide positive for blood-stage trophozoite *P. falciparum* or *P. vivax* parasites at any density for Thailand. Although clinical episodes were defined as a slide positive for blood-stage trophozoite *P. falciparum* parasites at any density with associated fever or fever-related symptoms for Gouye Kouly symptomatic episodes were too few to generate sufficient gametocyte data. All positive malaria cases were treated with appropriate antimalarial treatment according to the recommendation of the Malaria Division, Ministry of Public Health, as previously described [38]–[40], namely quinine until 1995 and then chloroquine in Dielmo and Ndiop and in Thailand mefloquine+primaquine for *P. falciparum* and chloroquine+primaquine for *P. vivax*. Sulfadoxine-pyrimethamine in conjunction with amodiaquine was used in Gouye Kouly, as this study occurred after a national policy change in 2004.

#### Asymptomatic episodes (active case detection)

Monthly systematic blood slides were taken from participating individuals from 1990–1998 and 1993–1998 in Dielmo and Ndiop respectively. In Gouye Kouly an intensive sampling schedule was implemented for 2005: prior to the rains in June and then every week for 8 weeks following the onset of the rains (first week of July). At each time point a thick blood smear was taken from all individuals. In the June sample and every two weeks from July, approximately 500 µL of blood were taken by finger prick from each individual in an EDTA microtainer (Sarstedt), of which 200 µL were mixed with in 1ml TRIzol® (Invitrogen), kept on dry ice and then frozen at −80°C for RNA extraction. Following DNA extraction and PCR amplification, all individuals' samples that were found to be positive for *P. falciparum* were then analysed for the presence of gametocytes by RT-PCR. The cohort was randomly divided into two groups (by household) such that half the cohort provided such a blood sample every week. Although there were insufficient gametocyte positive blood smears in this cohort, the few there were enabled us to validate the RT-PCR method. Two cross-sectional analyses in Suan Phung, Thailand carried out in 1995 and 2003 yielded insufficient gametocyte data from asymptomatic infections.

In all cases parasite positivity was established as follows. Thick and thin blood films were prepared and stained by 3% Giemsa stain. Blood films were examined under an oil immersion objective at ×1000 magnification by the trained laboratory technicians and 200 thick film fields were examined to count the number of asexual and gametocyte parasite stages. Parasite species were identified on thin films and asexual parasite densities (per µL) were calculated from thick film by establishing the ratio of parasites to white blood cells (WBC) and then multiplying the parasite count by 8,000, the average WBC count per µL of blood. Gametocyte densities per microlitre were estimated by multiplying by 4 the count per 200 microscope fields; the average number of WBCs per field being approximately 10, thus generating 2000 WBCs per 200 fields and thus representing a quarter of a microlitre. The minimum detectable gametocyte density is thus estimated to be 4 per µL.

### Gametocyte data

#### Blood smears

Four gametocyte traits were considered: (i) gametocyte positivity (i.e. prevalence), (ii) cumulative gametocyte positivity, (iii) gametocyte density and (iv) maximum gametocyte density for an individual. Here, “trait” is applied in a very loose sense, and does not imply that any genetic influence on these “traits” is only resulting from the human genome. Gametocyte positivity was defined as the proportion of parasite positive infections that also carried gametocytes and thus addressed the tendency to produce gametocytes during each infection. The cumulative gametocyte positivity likewise addresses this tendency, but sums over the number of infections an individual has experienced (and therefore opportunity to carry gametocytes). In the epidemiological analyses these two traits are the same; however in the heritability analyses they are treated differently (see *Data analyses* below). In addition to considering all gametocyte densities, we analysed the individual's maximum gametocyte density because transmission to mosquitoes is weakly associated with gametocyte density in some studies [45], [46], although low gametocyte densities are well known to also permit transmission to mosquitoes [13], [47], [48].

The duration of gametocyte carriage for a single infection in endemic settings can last up to 30 days [12], [49]. The longevity and infectivity of gametocytes have been shown to persist for 3 weeks following chloroquine treatment of clinical cases [50]. To increase the probability that only independent symptomatic or asymptomatic episodes (of gametocyte production) from the same individual are considered, consecutive samples with blood-stage malaria parasites of the same species within 30 days were excluded. Mixed parasite species infections were also excluded. It is likely our sampling approach missed some episodes of gametocytaemia, and thus underestimated prevalence.

#### PCR and RT-PCR for *P. falciparum* gametocyte detection

DNA was extracted from all samples from Gouye Kouly using the standard phenol-chloroform extraction method and DNA amplified using the ssrRNA gene nested PCR method of Snounou *et al.*, 1993 [51]. RNA extraction was then performed from the TRIzol® (Invitrogen) conserved sub-samples of those found positive. RNA was extracted using TRIzol® (Invitrogen), following the protocol recommended by the manufacturer. The extracted RNA was directly analysed or stored at −80°C.

For the RT-PCR, “*Plasmodium falciparum* meiotic recombination protein DMC1-like protein” gene (AF356553) was selected because it is exclusively expressed in gametocytes [52] and contains introns. Primers were thus selected spanning an exon-exon junction, amplifying a 101 bp segment, in the middle of which a probe was designed, using Primer3 software [53]. Primer sequences were: forward primer GAM8_F 5′ ATATCGGCAGCGAAAATGTGT 3′; reverse primer GAM8_R 5′ GACAATTCCCCTCTTCCACTGA 3′ and probe GAMPRO 5′ (6-Fam)TGCCCTTCTCGTAGTTGATTCGATTATT(BHQ1) 3′. cDNA was synthesised and the reaction primed with GAM8_R. Briefly 8 µL of extracted RNA was mixed with buffer, dNTPs (final concentration 1mM), RNase-free water, AMV Reverse transcriptase (20U; Promega) and Ribonuclease inhibitor (20U; Promega). Amplification cycle conditions were: 10 min. at 65°C, 60 min. at 42°C, 5 min. at 95°C. Quantification of cDNA was carried out using a fluorescent probe assay. Briefly 2 µL of synthesised cDNA was mixed with 2× mastermix (ABGene), GAM8_R (final concentration: 400nM), GAM8_F (final concentration: 400nM), GAM8_PRO (final concentration: 300nM) and sterile water. The reaction was analysed with a Rotor Gene® real-time PCR machine (Corbett Research). Each sample was analysed in triplicate. A dilution series containing 1000, 100, 10, 1 and 10^−1^ gametocytes/µL was used. This RT-PCR methodology had previously been validated using *in vitro* parasite cultures, and its specificity for detection of gametocytes *in vivo*, and not asexual parasites, demonstrated in a sample of 47 individuals presenting with clinical falciparum malaria in Madagascar (ethics S4 and Supplementary material S1).

### Genotyping of HbS and α-globin 3.7 deletion mutations

#### HbS – PCR-RFLP (Senegal)

Following DNA extraction, a 559 bp fragment covering codon 6 of the β–globin gene (*HBB*) gene was amplified by PCR using the primers HbS_F: 5′-AGGGGAAAGAAAACATCAAGGGTC-3′ and HbS_R: 5′-ATAAGTCAGGGCAGAGCCATCTAT-3′. The amplification reaction was carried out using 5 µL of DNA in a reaction volume of 15 µL composed of MgCl_2_ [2.5mM], dNTPs [1mM], each primer [1µM], 1.5 µL PCR buffer (Qbiogene) and 0.04 µL Taq (Qbiogene). Amplification cycle conditions were: 4min at 94°C, and then 35 cycles of 30sec at 94°C, 30sec at 65°C, and 30sec at 72°C, with a final extension phase of 10 min. at 72°C. The amplified fragment (5 µL of DNA) was then digested by restriction enzyme Dde I (2U) in a reaction volume of 15 µL containing 1.5 µL 10× Buffer. Wildtype *HBB* yields 6 fragments of 201+97+89+88+50+37 base pairs, whereas HbS mutation yields 5 fragments of 298+89+88+50+37 bp.

#### α-globin 3.7 deletion

Following DNA extraction, we used the PCR multiplex protocol of Chong et al. (2000) [54] to detect the presence of the α-globin 3.7 deletion. Primers α2/3.7-F and α2-R amplify a 1800 bp fragment covering the α2-globin gene. The sequence corresponding to primer α2-R is lost with the α-globin 3.7 deletion. A third primer, 3.7/20.5-R, is located 3′ of the α1-globin gene and allows amplification of fragments of 2022/2029 bp in the presence of the α-globin 3.7 deletion. Primer sequences are α2/3.7-F: 5′-CCCCTCGCCAAGTCCACCC-3′, 3.7/20.5-R: 5′-AAAGCACTCTAGGGTCCAGCG-3′ and α2-R: 5′-AGACCAGGAAGGGCCGGTG-3′. The amplification reaction was carried out using 5 µL of DNA in a reaction volume of 15 µL composed of MgCl_2_ [1mM], dNTPs [300nM], primer α2/3.7-F [0.4µM], primer 3.7/20.5-R [0.8µM], primer α2-R [0.1µM], 3 µL PCR buffer HotStar (Qiagen), 3 µL PCR buffer Q (Qiagen) and 0.04 µL HotStar Taq (Qiagen). Amplification cycle conditions were: 15min. at 98°C for enzyme activation and DNA denaturation, and then 50 cycles of 45sec at 98°C, 1 min. 15sec at 65°C, and 2 min. 30sec at 72°C, with a final extension phase of 5 min at 72°C. The α-globin 3.7 deletion yields fragments of 2022/2029 bp and the intact α2-globin gene 1800 bp.

### Data analyses

#### Epidemiological data analyses

Table 1 gives a summary of the samples analysed. Statistical analyses and model fitting were conducted using the statistical package Genstat 7.1 [55]. For each site, all individuals in the study protocol were included in the analyses, irrespective of whether their family structure was known. Factors influencing the maximum gametocyte density of either *P. falciparum* or *P. vivax* were analysed by fitting a Generalized Linear Model (GLM) with a Poisson error structure (loglinear regression). For gametocyte traits with repeated measures for the same individual (i.e. gametocyte positivity and gametocyte density), a Generalized Linear Mixed Model (GLMM) was fitted with individual person as a factor in the random model. For analysis of the gametocyte positivity rate, a binomial error structure was implemented (thus a logistic regression). Explanatory factors included date, which was classified annually by semester, reflecting the transmission seasons and hereon denoted season. Additional factors were gender and age factored initially into eight groups (<1, 1–4, 5–9, 10–14, 15–24, 25–39, 40–59 and 60+ years of age); if age was overall significant in the minimum adequate model, age groups were combined when not significantly different as ascertained by t-test and the final statistical model applied. A dispersion parameter was estimated by the deviance method, because the data were over-dispersed; initial model fitting with a dispersion parameter of 1 (for binomial and poisson error structures) yielded residual deviance much larger than the residual degrees of freedom. F-statistics in the GLM and Wald statistics, which approximate to a χ^2^ distribution, in the GLMM were established. In the analyses of maximum gametocyte density, the number of gametocyte density data points per individual was used as a weight.

**Table 1:** Summary of samples used in epidemiological and genetic analyses.

	Site	Dielmo		Ndiop		Gouye Kouly	Suan Phung	
		Symptomatic	Asymptomatic	Symp.	Asymp.	Asymp.	Symp. *PF*	Symp. *PV*
**Gametocyte positivity**								
Epidemiological analyses								
	Data points	1168	2710	1226	2063	101	1796	978
	Individuals	239	343	313	379	79	949	517
Genetic analyses	Individuals	236	335	310	364	77	859	470
	Independent families	9	10	17	19	8	188	136
**Gametocyte density**								
Epidemiological analyses								
	Data points	201	1096	180	578		84	323
	Individuals	109	280	125	246		80	230
Genetic analyses	Individuals	109	272	125	241		73	206
	Independent families	8	10	12	13		47	78

For epidemiological analyses, presented are the number of data points analysed for each trait, the corresponding number of individuals implicated and hence residual values generated. For genetic analyses, presented are the number of these individuals for whom pedigree information was available and thus the number of independent families for each trait in the heritability analyses.

The residual variance not explained by these “environmental” factors was generated. Because a non-normal error distribution was used, Pearson rather than standardized normal residuals were generated. For the gametocyte trait “cumulative gametocyte positivity rate”, the sum of the residuals per person was then calculated and used in the genetic analysis. For analysis of “gametocyte positivity” and “gametocyte density”, all residual values for any individual (who had repeated parasite density measures) were then used in the genetic analysis. Only residuals from individuals for whom family structure was available were then analysed for heritability.

#### Genetic and house data analyses

To determine the contribution of genetic factors to the “cumulative gametocyte positivity rate” and “maximum gametocyte density”, we evaluated the heritability (*h^2^*) by using the SOLAR software package (version 2.1.4) [56]. SOLAR performs a variance components analysis of family data that decomposes the total variance of the gametocyte traits into components that are due to genetic (polygenic) (*h^2^*), individual or environmental (*e^2^*) and house (*c^2^*) effects. We tested for a heritable human component in each gametocyte trait by comparing likelihood between the reduced model, where total variation is due to environmental variation only, and the full model where total variation is composed of environmental and genetic effects estimated from the genetic relationship coefficient of each pair of individuals. When the null hypothesis was rejected, heritability (*h^2^*) was then estimated as the percentage of genetic variance of the total. Although SOLAR can additionally incorporate measured covariates (e.g. explanatory variables), a normal distribution is assumed. For this reason we took into account the contribution of such variables in an initial statistical analysis (section above) and generated residual value for the gametocyte traits. The relative contribution of genetic factors to variation in the trait was then estimated by the heritability (*h^2^*), defined by the ratio of genetic variance component to the residual trait variance [56]. As several traits showed residual kurtosis of more than 0.8, *tdist* option, which creates an extra parameter in the model to describe the distribution of the trait, was applied in all analyses. An additive model, which is a general model, making no assumptions of the dominant or recessive nature of the gene, was used to avoid multiplying tests. For estimation of heritability, we used information from families that had at least 2 members with the traits of interest.

Household can confound the estimation of the genetic contribution to a trait, because related individuals often live in the same house and therefore not only experience a similar level of overall exposure to parasites, but also are potentially exposed to more genetically related parasites. This latter may be especially important given the known genetic variation in the parasite gametocyte production [35]. A household or shared environment effect can be added by an additional variance component with a coefficient matrix (H) whose elements (house) are 1 if the relative pair shares the same environmental exposure or 0 otherwise. Genetic effect (i.e. heritability) is estimated using matrix of correlation coefficients for identity by descent (IBD) allele sharing in various types of family relative pairs, whose elements (phi2) provide the predicted proportion of genes of the whole genome that a pair of individuals share at least 1 allele [56]. In SOLAR we first included the house effect in the model. If the house effect was not significant (p value >0.05), we excluded it from the model for estimation of heritability.

For the gametocyte traits for which there were multiple residual values (i.e. “gametocyte positivity” and “gametocyte density”), we evaluated heritability by using the classic repeated measures model (from the “animal model”) [57], [58], where a permanent environmental effect is created for each individual. Thus the following model is fitted: y = [X*b*]+Z*a*+Z*pe*+Z*h*+*e* where y is the residual parasite density value, *b* is the fixed effects vector (here already taken into account in the first statistical analysis), *a* is the additive genetic effects vector, *pe* is the permanent environment effects vector (of each individual), *h* is the common house effect vector and *e* is the residual effects vector; X is the design matrix relating observations to fixed effects and Z are design matrices relating observations to random effects. The model was fitted using ASREML vers. 2 [57]. The total trait variance is therefore V_P_, which is partitioned into V_A_, additive genetic variance, V_PE_, variance due to permanent environmental effects, V_H_, common-house variance and V_R_, residual variance. Heritability (*h^2^*) is again V_A_/V_P_.

## Results

Table 2 presents a summary of the gametocyte data per infection type and study cohort. From 1990–1998 in Dielmo, there were 1,168 symptomatic *P. falciparum* episodes by 239 individuals; by microscopy, 201 (17.2%) of these infections from 109 individuals had gametocytes. The mean gametocyte density (excluding zeros) was 18.4/µL (SE 2.4, range 4–208). During the same time frame, there were 2,710 observations of asymptomatic *P. falciparum* infections in 343 individuals; 1,096 of these infections (40.4%) from 280 individuals had gametocytes. The mean gametocyte density was 37.2/µL (SE 5.2, range 4–3,588). From 1993–8 in Ndiop, there were 1226 symptomatic *P. falciparum* episodes by 313 individuals; by microscopy, 180 (14.7%) of these infections from 125 individuals had gametocytes. The mean gametocyte density was 69.3/µL (SE 15.8, range 4–1,984). During the same time frame, there were 2,063 observations of asymptomatic *P. falciparum* infections in 379 individuals; 578 of these infections (28%) from 246 individuals had gametocytes. The mean gametocyte density was 22.2/µL (SE 3.1, range 4–908). From June–August 2005 in Gouye Kouly, there were 101 independent *P. falciparum* positive asymptomatic observations in 79 individuals; there was one observation for 58 individuals, two observations for 20 and three for one individual. 79 infections (78%) had gametocytes, as detected by RT-PCR; density was not, however, ascertained in the RT-PCR. From 1999–2004 in Suan Phung, there were 1,796 symptomatic *P. falciparum* episodes presented by 949 individuals; by microscopy, 84 (4.7%) of these infections from 80 individuals had gametocytes. The mean gametocyte density was 284.5/µL (SE 62.8, range 1–3,480). During the same period, there were 978 observations symptomatic *P. vivax* episodes presented by 517 individuals; 323 of these infections (33%) from 230 individuals had gametocytes. The mean gametocyte density was 648/µL (SE 63.5, range 16–11,280).

**Table 2:** Data summary of the number of asexual parasite positive infections, the number of individuals having at least one asexual parasite positive record, the median and range of the number of asexual parasite positive records per person, the number of asexual parasite positive infections that had gametocytes, the number of individuals having at least one gametocyte positive record, the median and range of the number of gametocyte positive records per person.

Site	Infection	Total #	# individuals	Median (range)	Total #	# individuals	Median (range)
	Type	parasite positive observations	parasite positive	# parasite positive per person	gametocyte positive observations	gametocyte positive	# gametocyte positive per person
Dielmo	Symp	1168	239	3 (1–23)	201	109	1 (1–5)
	Asymp	2710	343	7 (1–22)	1096	280	3 (1–15)
Ndiop	Symp	1226	313	3 (1–13)	180	125	1 (1–5)
	Asymp	2063	379	5 (1–20)	578	246	2 (1–11)
Gouye Kouly	Asymp	101	79	1 (1–3)	79	49	1 (1–2)
Suan Phung	Symp PF	1796	949	1 (1–12)	84	80	1 (1–2)
	Symp PV	978	517	1 (1–11)	323	230	1 (1–6)

Symp – symptomatic infection; Asymp – asymptomatic infection. PF - *P. falciparum*; PV – *P. vivax*. # - number.

The genotype frequencies of AS (HbS heterozygote) were 9.9% (N = 46 of 466 individuals successfully genotyped) in Dielmo, 13.6% (N = 67 of 493 individuals successfully genotyped) in Ndiop and 7.1% (N = 21 of 295 individuals successfully genotyped) in Gouye Kouly. There were two SS (HbS homozygote) in Dielmo and none in either Ndiop or Gouye Kouly. The genotype frequencies of the heterozygote alpha-globin 3.7 deletion were 18.1% (N = 75 of 415 individuals successfully genotyped) in Dielmo, 30.2% (N = 132 of 437 individuals successfully genotyped) in Ndiop; the alpha-deletion was not typed in Gouye Kouly. The homozygote alpha-deletion genotype frequencies were 1.2% in Dielmo and 1.8% in Ndiop. In Suan Phung (Thailand), the heterozygote alpha-globin 3.7 deletion genotype frequency was 15.8% (N = 139 of 881 individuals successfully genotyped) and the homozygote genotype frequency was 1.02% (N = 9 individuals). Table 3 presents the genotype frequencies of alpha and beta globin gene mutations for which there were corresponding gametocyte data and hence used in the statistical analyses.

**Table 3:** Genotype frequencies for sickle cell mutation (HbS) and alpha-globin 3.7 deletion.

		Sickle cell mutation			alpha globin - 3.7deletion		
Site	Infection type	AA	AS	SS	Wildtype	heterozygote	homozygote
Dielmo	Symp	272	36	1	215	49	2
	Asymp	312	33	1	237	51	4
Ndiop	Symp	251	36		176	69	5
	Asymp	331	48		222	95	7
Gouye Kouly	Asymp	73	6		ND	ND	ND
Suan Phung	SympPF				318	63	5
	SympPV				190	26	4

HbS is not present in Suan Phung (Thailand); HbE and other beta-globin mutations were found very infrequently and are not indicated. Symp – symptomatic infection; Asymp – asymptomatic infection. PF - *P. falciparum*; PV – *P. vivax*. ND – not determined.

**Figure 1:**
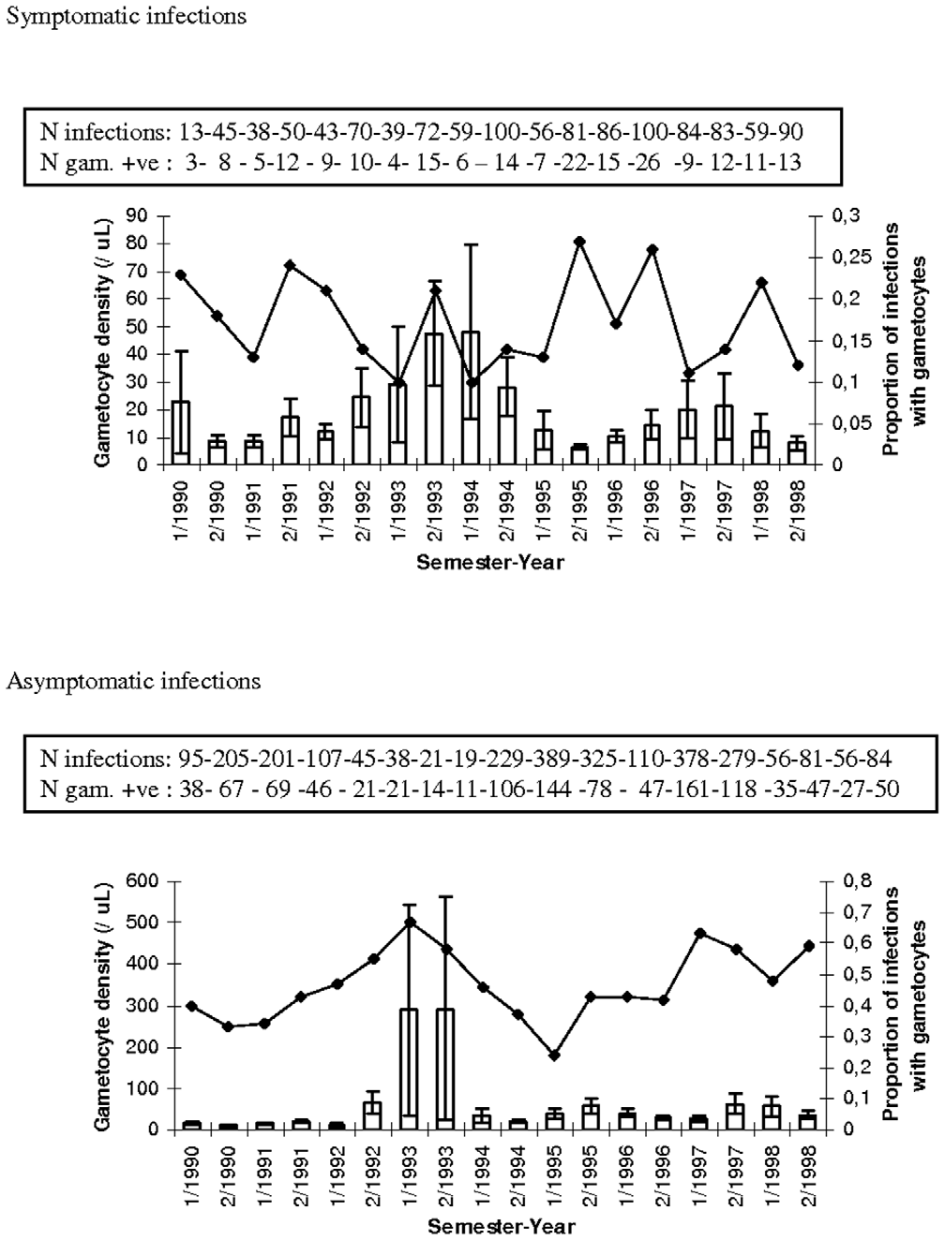
Gametocyte prevalence (line plot) and density (histogram) in symptomatic and/or asymptomatic infections by semester-year in Dielmo. 1/“year” indicates the first semester and 2/“year” the second semester of each year. Shown are means and SE for gametocyte density. Given in the boxes are the corresponding number of infections of *P. falciparum* and the number of these that were positive for gametocytes (and hence used to calculate the gametocyte densities).

**Figure 2 pone-0011358-g002:**
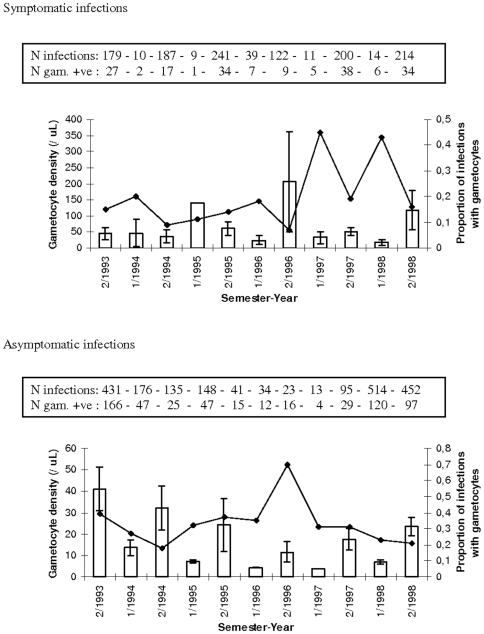
Gametocyte prevalence (line plot) and density (histogram) in symptomatic and/or asymptomatic infections by semester-year in Ndiop 1/“year” indicates the first semester and 2/“year” the second semester of each year. Shown are means and SE for gametocyte density. Given in the boxes are the corresponding number of infections of *P. falciparum* and the number of these that were positive for gametocytes (and hence used to calculate the gametocyte densities).

Table 4 presents the summary of the epidemiological analyses showing significance level and percentage of variation in *P. falciparum* (Pf) and *P. vivax* (Pv) gametocyte traits explained by environmental variables and the two genetic mutations (HbS and alpha-globin 3.7 deletion). Age, season and asexual parasite density had a consistently significant impact on gametocytes. For gametocyte positivity, the impact of these factors was, however, small. The proportion of *P. falciparum* infections carrying gametocytes decreased with increasing age and asexual parasite density. In Ndiop, individuals of ten years and older had reduced odds of carrying gametocytes whether in symptomatic (Odds Ratio = 0.42 [95%Confidence Intervals 0.28–0.56]) or asymptomatic infections (OR = 0.56 [95%CI 0.43–0.68]). Similarly, in Dielmo 10+ year old individuals similarly had lower odds of carrying gametocytes when infected, as compared to the youngest age group (0–4 years) whether in symptomatic (OR = 0.36 [95%CI 0.26–0.47]) or asymptomatic infections (OR = 0.17 [95%CI 0.08–0.25]). In Suan Phung there was also significantly lower odds of carrying *P. falciparum* gametocytes for the older (>15 years) age group (OR = 0.32 [95%CI 0.22–0.42]). *P. vivax* gametocyte positivity increased with asexual parasite density, but was not affected by age. Both age and asexual parasite density were inversely correlated to gametocyte density. Age and especially season explained a large amount of the observed variation. However, as shown in Figure 1–3, variation in gametocyte traits was as great, if not greater, across years than between seasons, with one exception: the increase in gametocyte density during the rainy season (season 2 of each year) in asymptomatic infections in Ndiop, where transmission is highly seasonal (Fig. 2).

**Figure 3:**
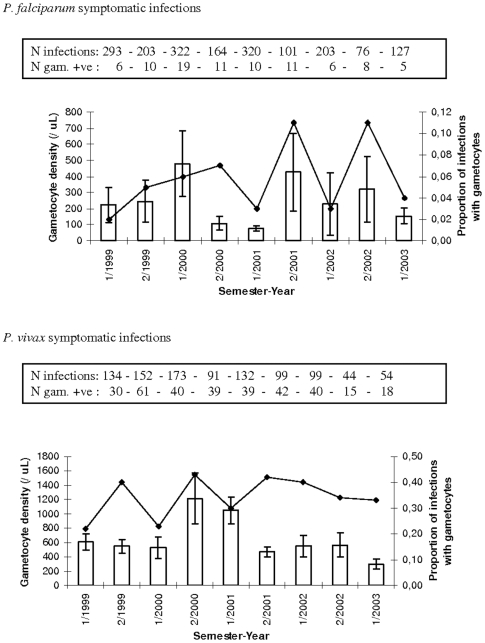
Gametocyte prevalence (line plot) and density (histogram) in symptomatic infections by semester-year in Suan Phung. 1/“year” indicates the first semester and 2/“year” the second semester of each year. Shown are means and SE for gametocyte density. Given in the boxes are the corresponding number of infections of *P. falciparum* or *P. vivax* and the number of these that were positive for gametocytes (and hence used to calculate the gametocyte densities).

**Table 4:** Summary of epidemiological analyses showing percentage of variation in *P. falciparum* (Pf) and *P. vivax* (Pv) gametocyte traits explained by environmental variables and two human genetic mutations.

Gametocyte Positivity											
Site	Infection type	Age		Date		Asexual parasite density		HbS		α-globin 3.7 deletion	
		%	P	%	P	%	P	%	P	%	P
Dielmo	Symptomatic	0.3	0.0017	0	0.16	0.3	0.015	0.3	0.047	0	0.90
	Asymptomatic	3.3	<0.001	2.4	<0.001	2.7	<0.001	<0.1	0.021	0	0.24
Ndiop	Symp	2.3	<0.001	2.3	0.007	2.7	<0.001	1.4	<0.001	0	0.92
	Asymp	0.6	0.004	2.2	<0.001	0	0.52	0.2	0.016	0	0.91
Gouye Kouly	Asymp	0	0.45	4.5	<0.001	10.4	<0.001	0	0.23	ND	ND
Suan Phung	Symp PF	3.4	<0.001	5.7	<0.001	2.0	<0.001	NA	NA	0	0.47
	Symp PV	0	0.37	1.5	0.002	5.3	<0.001	NA	NA	0	0.26

Gametocyte density											
Site	Infection type	Age		Date		Asexual parasite density		HbS		α-globin 3.7 deletion	
		%	P	%	P	%	P	%	P	%	P
Dielmo	Symp	1.9	0.037	20.1	<0.001	9.2	0.004	2.4	0.026	0	0.93
	Asymp	16.3	<0.001	20.8	<0.001	0	0.08	0	0.56	0	0.44
Ndiop	Symp	9.6	<0.001	8.0	<0.001	1.0	0.037	0	0.92	0	0.26
	Asymp	2.5	0.003	12.6	<0.001	3.4	<0.001	0	0.88	0	0.29
Suan Phung	Symp PF	12.6	0.002	0	0.23	6.2	0.005	NA	NA	0	0.97
	Symp PV	5.6	0.011	0	0.11	1.2	0.001	NA	NA	0	0.64

Maximum gametocyte density											
Site	Infection type	Age		Date		Asexual parasite density		HbS		α-globin 3.7 deletion	
		%	P	%	P	%	P	%	P	%	P
Dielmo	Symp	9.4	<0.001	37.2	<0.001	7.1	<0.001	0	0.33	0	0.85
	Asymp	26.3	<0.001	35.8	<0.001	0	0.21	0	0.37	0	0.38
Ndiop	Symp	14.2	<0.001	15.9	0.002	0	0.32	0	0.062	0	0.13
	Asymp	4.5	<0.001	28.2	<0.001	2.2	0.006	0	0.067	0	0.19
Suan Phung	Symp PF	0	0.09	0	0.13	0	0.94	NA	NA	0	0.44
	Symp PV	0	0.11	0	0.15	4.4	<0.001	NA	NA	0	0.22

In parentheses, p is the p-value, otherwise p<10^−3^; ND – not done. NA – not applicable; the HbS mutation was not found in Suan Phung (Thailand). Age: 2 groups in Ndiop, 0–9 & 10+ years old; 3 groups in Dielmo: 0–4, 5–9, 10+; age is a continuous variable in Gouye Kouly; 2 groups in Suan Phung 0–14 & 15+. Date: by season (semester-year) in Ndiop, Dielmo and Suan Phung, and by month (3) in Gouye Kouly. Because of low numbers of homozygote mutations in *HBB* (beta-globin) and *HBA* (alpha-globin), these groups were combined with heterozygote mutation group and compared with wildtype (See Table 3). Symp – symptomatic infection; Asymp – asymptomatic infection. PF - *P. falciparum*; PV – *P. vivax*.

There was no impact of the alpha-globin 3.7 deletion (comparing wildtype with heterozygote plus homozygote deletion groups) on gametocytes in any study site (Table 4). By contrast, there was a significant effect of HbS (heterozygote plus homozygote) on gametocyte positivity. In both symptomatic and asymptomatic infections in Dielmo and Ndiop, there was a greater proportion of infections with gametocytes in individuals carrying the sickle cell mutation (Dielmo Symptomatic OR 1.99 [95%CI 1.35–2.63]; asymptomatic OR: 1.59 [95%CI 1.12–2.05]; Ndiop Symptomatic OR 1.53 [95%CI 1.09–1.97]; asymptomatic OR: 1.67 [95%CI 1.25–2.09]). HbS was also associated with an increase in gametocyte density in Dielmo, explaining 2.4% of the variation in this trait.

### Estimation of heritability and house effect

The estimated human genetic contribution (*h^2^*) to gametocyte production is given in Table 5. In all three studies of *P. falciparum* asymptomatic infections, there was apparent heritability in cumulative and overall gametocyte positivity. Heritability was moderate in Dielmo and Ndiop (15.6% SE 8.0 & 16.3% SE 8.0) but high in Gouye Kouly (57.1% SE 24.4) for cumulative gametocyte positivity. Similar values were obtained for per infection gametocyte positivity (Dielmo 21.4% SE 10.1; Ndiop 19.3% SE 8.4; Gouye Kouly 48.2% SE 22.1). There was no heritability for symptomatic infections carrying gametocytes of either *P. falciparum* or *P. vivax*. Our estimate of heritability of (cumulative) gametocyte positivity was not significantly altered by taking into account the effect of HbS (Table 5). There was no human genetic contribution to gametocyte density detected in our analysis. In our model output, there were no apparent effects of house on any of the gametocyte traits.

**Table 5:** Estimated heritability of the proportion of infections that carry gametocytes (cumulative over all infections for an individual – see *Data analyses*).

Site	Infection type	prior adjustment for environmental effects			prior adjustment for environmental and HbS effects		
		N	h^2^ (SE)	P	N	h^2^ (SE)	P
Dielmo	Symp	301	0.06 (0.08)	0.22		-	-
	Asymp	335	**0.156 (0.08)**	**0.0087**	311	**0.174 (0.091)**	**0.007**
Ndiop	Symp	286	0.006 (0.072)	0.47		-	-
	Asymp	364	**0.163 (0.08)**	**0.006**	362	**0.135 (0.08)**	**0.018**
Gouye Kouly	Asymp	77	**0.571 (0.244)**	**0.007**		-	-
Suan Phung	Symp PF	859	0.07 (0.06)	0.099		-	-
	Symp PV	470	0.03 (0.10)	0.37		-	-

The significant effects of environmental factors (and additionally sickle cell mutation) (Table 4) are accounted for by initial analyses and then the unexplained residual variation is analysed for heritability. Note that HbS was not found to be significant in the initial analyses in Gouye Kouly and thus not adjusted for. HbS – sickle cell mutation. Symp – symptomatic infection; Asymp – asymptomatic infection. PF - *P. falciparum*; PV – *P. vivax*.

**Figure 4:**
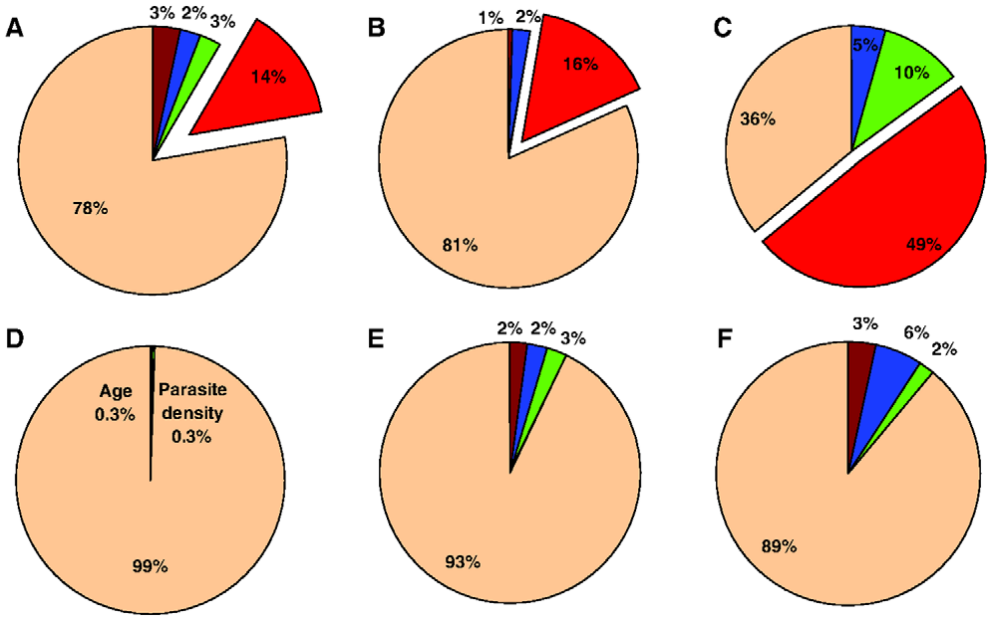
Proportion of variation explained by genetic heritability and environmental factors found to have a significant effect on *P. falciparum* gametocyte positivity (**Table 4** & **5**). (A) Asymptomatic infections, Dielmo (B) Asymptomatic infections, Ndiop (C) Asymptomatic infections, Gouye Kouly (D) Symptomatic infections, Dielmo (E) Symptomatic infections, Ndiop (F) Symptomatic infections, Suan Phung. Colour coding: Brown, age; Blue, date; Green, asexual parasite density; red, human genetics; beige, other.

We have sought to partition the total variation in the number of infections that carry gametocytes into its genetic and environmental components (Tables 4 & 5 and Figure 4). Of particular note are the moderate to high genetic contributions to gametocyte positivity (both cumulative and individual) in asymptomatic infections but lack of genetic contribution in symptomatic infections in the estimates generated by our model. Season consistently contributed to gametocyte positivity in the sites of seasonal transmission irrespective of infection type. Strikingly, no single factor explained any significant variation (i.e. >1%) in gametocyte positivity in symptomatic infections in Dielmo (Table 4).

## Discussion

This study sought to evaluate the extent of human genetic contribution to the prevalence and density of gametocytes during asymptomatic and symptomatic infections of *P. falciparum* across a range of epidemiological settings. We have presented good evidence for a significant human genetic contribution to gametocyte prevalence in asymptomatic infections. Our model estimated consistent, moderate heritability in the tendency to carry gametocytes during asymptomatic infections, which became considerably stronger when more sensitive methods of detection identified more gametocyte carriers. By contrast, we found no evidence of a human genetic contribution to gametocyte production in symptomatic infections.

The most likely explanation for the apparent differences in heritability of gametocyte production between asymptomatic and symptomatic infections is that individuals with symptomatic episodes will come for treatment prior to the production of gametocytes in our study sites. *P. falciparum* gametocytes require 7–10 days to mature and become patent in a thick blood smear [59]. Moreover, there appears to be a variable degree of tolerance to parasite density prior to eliciting symptoms [23], [26], [39], [60], [61]. Variation in the speed of symptomatic reaction to the infection may therefore further increase variation in gametocyte traits at clinical presentation. The absence of a consistent contribution of asexual parasite density to gametocyte production in symptomatic infections supports this hypothesis. In these respects, our study is therefore limited in its capacity to generate robust symptomatic gametocyte traits that reflect reality.

The absence of a human contribution to *P. vivax* gametocyte traits here and in a previous study in Sri Lanka [62] can not, unlike *P. falciparum*, be explained by the slow development of gametocytes. *P. vivax* gametocytes develop at the same speed as asexual stages and are produced simultaneously. Indeed, there was a positive relationship between asexual parasite density and *P. vivax* gametocyte traits. Previously a human genetic contribution to *P. vivax* asexual parasite density was identified in this population [58] and therefore *P. vivax* gametocyte production may be intimately linked to asexual parasite density. Further data on gametocyte production in asymptomatic infections is, however, required to resolve the potential for there to be a human genetic contribution to gametocyte positivity that is independent of asexual parasite density.

Differences in gametocyte prevalence rates among sympatric ethnicities have been noted previously, suggestive of human genetic influence on gametocyte production [20]–[22]. A previous study to examine heritability in gametocyte traits, however, found no heritability [62]. That study was carried out in a population where the transmission intensity was similar to our Thai study site and thus most likely concern mainly symptomatic infections. Previously identified risk factors for gametocyte carriage have concentrated on symptomatic episodes and identified anaemia [6] and hyperparasitaemia [7], as well as an effect of certain anti-malarial drugs such as chloroquine [3], [4]. These factors are unlikely to be important for asymptomatic infections, although a degree of anaemia, or more broadly haematological insult, may occur in chronic asymptomatic infections [63]. Two candidate genes, beta-globin and alpha-globin, were chosen because of their recognized impact on malaria parasite infection [16], [17], [19], [64], [65] and determinant role in anaemia [18], [36].

In our study, only HbS was found to have an impact in the epidemiological analyses, being associated with increased gametocyte positivity and density. HbS explained 2.4% of the variation in gametocyte density in symptomatic infections, a value similar to the estimated protective effect afforded by HbS against clinical disease [60]. Increased gametocyte production has been observed *in vitro* using reticulocytes from anaemic patients, including those suffering from sickle cell disease [5]. Accounting for the effect of this gene on gametocyte positivity yielded no significant change in the extent of heritability, however, suggesting that other co-factors are required. The role of HbS in eliciting gametocyte production requires further study, especially as *in vivo* transmission studies have suggested that gametocytes from individuals with sickle cell mutation are more infectious to mosquitoes, even at similar gametocyte densities [66]. Moreover, a very recent study did indeed observe increased gametocyte carriage in individuals with HbC [31].

Our epidemiological analyses highlight consistent pertinent factors, namely age, asexual parasite density and season, having an impact, albeit slight, on *P. falciparum* gametocyte prevalence. Season has previously been identified as having an impact on gametocyte production, with notably increased gametocyte prevalence during the transmission season [67]–[71]. This seasonal increase is most clearly observed for gametocyte density in Ndiop in asymptomatic infections, but less clear in the other studies. Such an increase in the gametocyte reservoir in the asymptomatic population will have significant impact on parasite transmission and the underlying biology needs to be explored. The weakly inverse relationship of gametocyte prevalence and asexual parasite density is consistent with the dichotomous developmental trade-off whereby an asexual parasite must commit to the production of either asexual or gametocyte stages. It should be emphasized that asexual parasite density at the time of measurement of the gametocyte phenotype is not the same as that occurring at the time of gametocyte developmental conversion, which occurs seven or more days earlier. This abnegates unequivocal conclusions on the role of asexual parasite density in gametocyte production with our data.

The impact of age on both gametocyte prevalence and density observed in our analyses may to some extent reflect the lower asexual parasite densities in older age groups resulting from the acquisition of immunity [68]. This would result in a smaller source of asexual parasites for gametocyte production and hence a reduced gametocyte density that makes their detection more difficult. The effect of age may additionally be the result of anti-gametocyte immune responses [72]–[75], but whose significance remains uncertain. It has been noted that when gametocytes are present in older age groups, their densities, relative to the asexual parasite density whence they arise, are generally increased [74]. This is consistent with the known influence of both specific and non-specific anti-asexual parasite immune mechanisms on the rate of conversion from asexual to gametocyte stage parasites [76]–[78]. The absence of a human contribution to gametocyte density, however, argues against genetic variation in such immune mechanisms playing a significant role in determining gametocyte positivity.

In all studies addressing heritability of a quantifiable trait, the robustness of the result is dependent on the accuracy with which the trait is defined and measured. Gametocyte traits are complex traits that are likely to be influenced by the human, parasite and potentially even the mosquito, all within the context of the local actual and historical transmission intensity as well as local environmental heterogeneity. Specific drug treatment regimens may exert different selection pressures on the parasite populations and contribute to site-specific differences in parasite traits. Moreover, gametocytes are intimately linked to asexual parasites and decoupling *P. falciparum* gametocyte dynamics from asexual parasite dynamics is challenging, especially given the developmental time-lag of gametocytes and the sequestration of the asexual parasites that hinder accurate measure of density. In a first attempt, we have used very simple gametocyte traits and it is remarkable that, in our model, a consistent human genetic contribution was observed in the two sites that employed a comparable sampling protocol. Although the significantly increased value obtained in Gouye Kouly might be the result of the more sensitive gametocyte detection method, comparing heritability among populations is not meaningful because the genetic make-up of the human population and the environment (here including the parasite population) will differ among populations and even within the same population over time. Thus, whilst reproducibility of genetic effects in different populations is essential for validation, the precise heritability values given here must be taken with caution, not least because estimating heritability is fraught with confounding issues, most notably those associated with economic status and sharing a household, that are to some extent family-dependent (For a good discussion see the commentaries associated with [60]). In addition, there is in general some confusion over the actual meaning of heritability. In brief, significant heritability for gametocyte carriage suggests that there is a human genetic contribution for variation in this trait. It does not, however, mean that, in this study, between 16% (Dielmo and Ndiop) and 50% (Gouye Kouly) of gametocyte carriage is caused by human genes.

The results presented here provide sufficient evidence that more detailed and thorough genetical and epidemiological studies are worthwhile. Prospective epidemiological studies will provide an opportunity to generate novel and perhaps more epidemiologically pertinent gametocyte data. Our choice of excluding consecutive samples with blood-stage malaria parasites of the same species within 30 days of the first sample not only likely underestimates gametocyte carriage, especially that at a low density, but also fails to capture the functional true reservoir of infection. In particular, such duration of gametocyte carriage is of evident importance [15], as is the impact of multiplicity of infection of the same and of other parasite species [79], [80]. The existence of a significant human genetic contribution to gametocyte prevalence suggests that candidate gene and genome wide association approaches are now needed to identify the underlying biological processes that may explain this.

## Supporting Information

Ethics S1 Ethics approval for Dielmo and Ndiop.
(0.85 MB JPG)

Ethics S2 Ethical approval from Institut Pasteur Biomedical Research Committee and Ministry of Health Ethics Committee Senegal.
(0.62 MB PDF)

Ethics S3 Thai study site ethics approval.
(0.27 MB JPG)

Ethics S4 Ethical permission to carry out RT-PCR validation in field samples.
(0.21 MB PDF)

Supplementary Material S1 Validation experiments for gametocyte RT-PCR.
(0.18 MB DOC)
