# Supplementary figures and images for: Heritability of the Human Infectious Reservoir of Malaria Parasites
Source: PLoS One. 2010 Jun 29;5(6):e11358. doi: 10.1371/journal.pone.0011358 (PMC2894056; doi:10.1371/journal.pone.0011358)

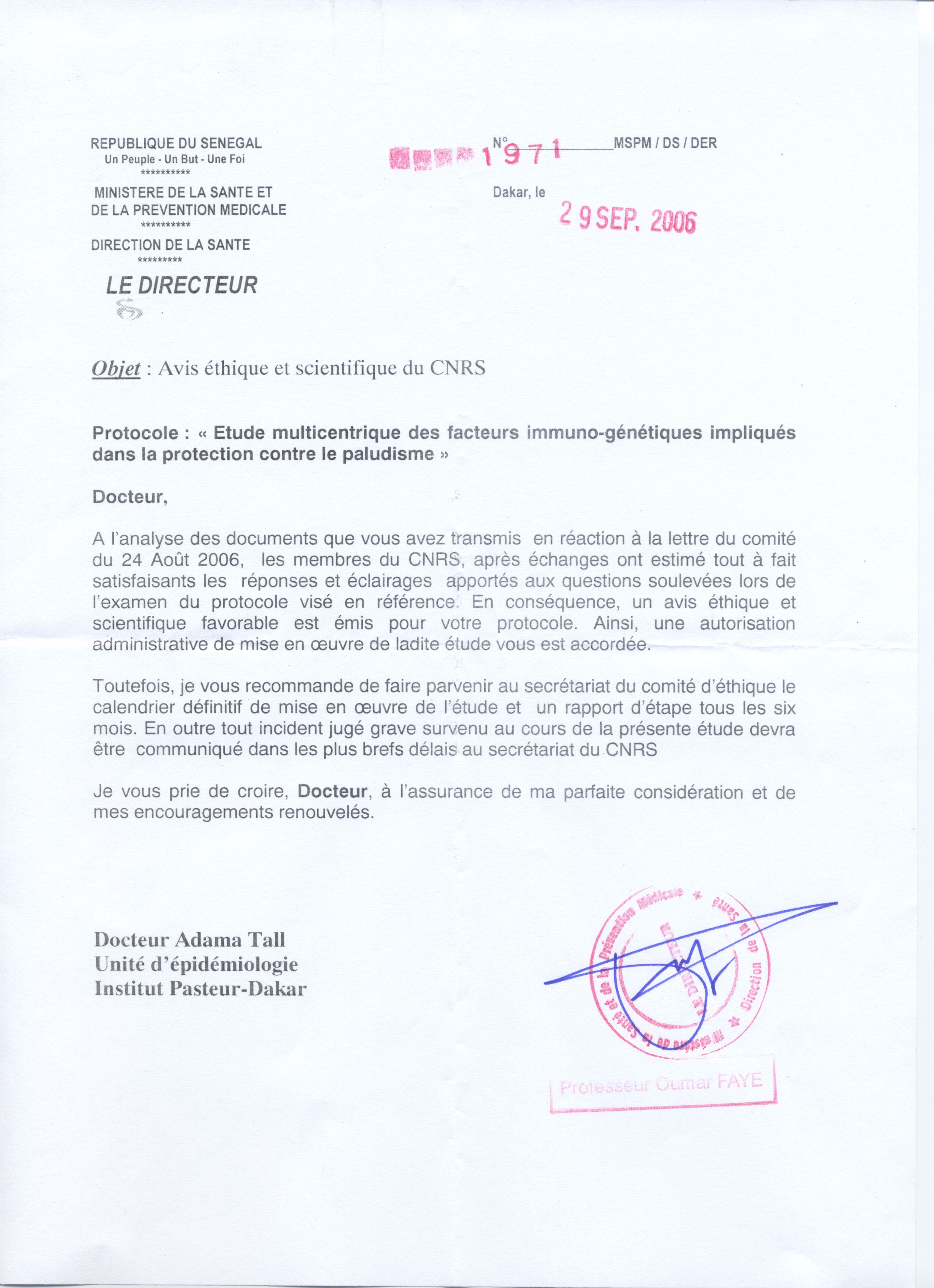

Supplement: Ethics S1 — Ethics approval for Dielmo and Ndiop. (0.85 MB JPG) [file pone.0011358.s001.jpg]

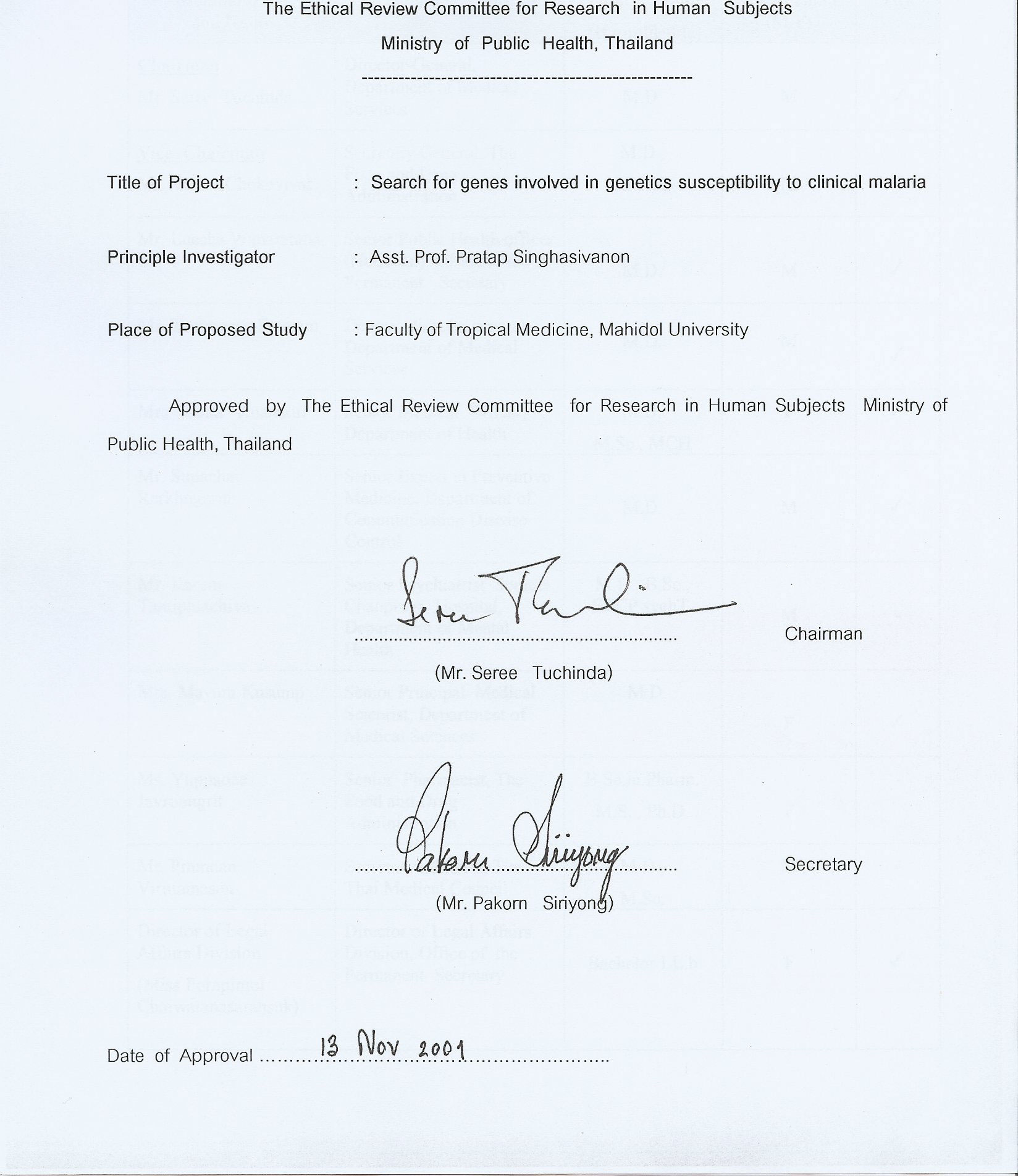

Supplement: Ethics S3 — Thai study site ethics approval. (0.27 MB JPG) [file pone.0011358.s003.jpg]
