# Supplementary material for: Heritability of the Human Infectious Reservoir of Malaria Parasites
Source: PLoS One. 2010 Jun 29;5(6):e11358. doi: 10.1371/journal.pone.0011358 (PMC2894056; doi:10.1371/journal.pone.0011358)
Supplement: Ethics S2 — Ethical approval from Institut Pasteur Biomedical Research Committee and Ministry of Health Ethics Committee Senegal. (0.62 MB PDF) [file pone.0011358.s002.pdf]

INSTITUT PASTEUR

Direction Médicale

Paris, le 01 juillet 2004

Docteur Richard PAUL
Institut Pasteur de Dakar
36, Avenue Pasteur
BP 220
DAKAR SENEGAL

Nos Réf. : JPL/JF/EdelaG/2004.016

Objet : Recherche biomédicale " *Evaluation du rôle immuno-mdulateur de la salive de moustique dans le développement de l'infection par P. falciparum chez l'homme et la transmission du parasite au moustique* "

AVIS DU COMITE DE RECHERCHE BIOMEDICALE

DEPOSE LE 11/06/2004	N° ENREGISTREMENT 2004.016	CATEGORIE ADMINISTRATIVE : 2A

Docteur,

Le Comité de Recherche Biomédicale du 22/06/2004 a donné à propos de votre dossier un avis de principe :

☒ **Favorable** (sous réserve des commentaires ci-joints)
☐ **Défavorable** (Cf. explications dans le document ci-joint)

De plus, et dans ces conditions,
demande de PROMOTORAT ASSURE PAR L'INSTITUT PASTEUR :

☐ **non applicable** ☐ **refusée** (Cf. explications dans le document ci-joint)
☒ **acceptée**

La participation du SPEB à cette recherche est acceptée en tant que Centre d'Investigation Clinique : ☐ Oui ☒ Non

Le CRVBm pourra assurer le suivi méthodologique et réglementaire de votre étude : ☐ Oui ☒ Non
Merci de prendre contact avec : Docteur Christine Sadorge : csadorge@pasteur.fr / poste 3826

ATTENTION :
Toute modification du protocole APRES acceptation du dossier doit être soumise à la Direction Médicale

Vous souhaitant bonne réception de la présente et dans l'attente des éventuels documents réclamés à faire parvenir à mon secrétariat, je vous prie d'accepter, Docteur, mes salutations distinguées.

Pr. Jean-Paul Lévy

P.J : Note d'information sur les documents juridiques et réglementaires nécessaires pour une recherche se déroulant en France

C.C : E. de la Goutte, A des Graviers, C. Sadorge, J. Fleury, P. Buffet, JP Saintouil

REPUBLIQUE DU SENEGAL
******
MINISTERE DE LA SANTE ET
DE PREVENTION MEDICALE
******
DIRECTION DES ETUDES, DE LA
RECHERCHE ET DE L AFORMATION

LE DIRECTEUR

N° 913 /MSPM/DERF/DER

Dakar, le 30 JUIL. 2004

Protocole « Evaluation du rôle immuno-modulateur de la salive de moustique dans le développement de l'infection par *P. falciparum* chez l'homme et la transmission du parasite au moustique »

<u>Objet</u> : Avis éthique et scientifique (Réunion 07 et 08 Juillet 2004)

Ref :V/L du 26 Juillet 2004

Docteur,

Les éléments d'informations que vous avez transmis au secrétariat du CNRS par l'entremise de la lettre visée en référence répondent d'une manière satisfaisante aux questions soulevées par le CNRS lors de sa réunion des 07 et 08 Juillet 2004.

En conséquence, le CNRS autorise la mise en œuvre de l'étude et souhaite un plein succès à vos travaux de recherche.

Je vous prie de croire, **Docteur**, à l'assurance de ma considération distinguée et à mes encouragements anticipés.

Dr
Richard PAUL
Laboratoire d'Entomologie Médicale
Institut Pasteur de Dakar,
36 Avenue Pasteur, Bp 220

Directeur

Dr Babacar DRAME
