# Supplementary material for: Heritability of the Human Infectious Reservoir of Malaria Parasites
Source: PLoS One. 2010 Jun 29;5(6):e11358. doi: 10.1371/journal.pone.0011358 (PMC2894056; doi:10.1371/journal.pone.0011358)
Supplement: Ethics S4 — Ethical permission to carry out RT-PCR validation in field samples. (0.21 MB PDF) [file pone.0011358.s004.pdf]

Nenard + Jocelyn

REPOBLIKAN'I MADAGASIKARA
Tanindrazana – Fahafahana – Fandrosoana

MINISTERE DE LA SANTE
ET DU PLANNING FAMILIAL

Le Ministre

N° 007 -SANPF/Le Ministre

AUTORISATION

Après consultation et avis favorable du Comité d'Ethique auprès du Ministère de la Santé, **l'Institut Pasteur de Madagascar est autorisé** à effectuer la recherche intitulée : **«Etude multicentrique, randomisée, comparative, d'efficacité et de tolérance de 5 schémas thérapeutiques : chloroquine, amodiaquine, association fixe sulfadoxine + pyriméthamine, combinaison amodiaquine+association fixe sulfadoxine+pyrimethamine et combinaison artesunate+amodiaquine dans le traitement de l'accès palustre simple biologiquement confirmé à Plasmodium falciparum »**.

Fait à Antananarivo, le 0 8 JAN. 2007

Le Ministre de la Santé et du Planning Familial

MINISTERE DE LA SANTE ET DU PLANNING FAMILIAL
REPOBLIKAN'I MADAGASIKARA

Dr. ROBINSON JEAN LOUIS
