# Supplementary material for: Heritability of the Human Infectious Reservoir of Malaria Parasites
Source: PLoS One. 2010 Jun 29;5(6):e11358. doi: 10.1371/journal.pone.0011358 (PMC2894056; doi:10.1371/journal.pone.0011358)
Supplement: Supplementary Material S1 — Validation experiments for gametocyte RT-PCR. (0.18 MB DOC) [file pone.0011358.s005.doc]

**Quantitative RT-PCR for the quantification of *Plasmodium falciparum* gametocytes in clinical samples**

Validation of RT-PCR protocol for “*Plasmodium falciparum* meiotic recombination protein DMC1-like protein” gene (AF356553)

**Sensitivity and specificity**

The technique was initially tested on a *Plasmodium falciparum* gametocyte-producing strain (clone 3D7), after which a field isolate was used. The following results are taken from an experiment in which a sample collected in Kompong Cham, Cambodia in 2006 was analysed with the protocol described in the main manuscript in order to validate the protocol and establish a gametocytaemia dilution series to analyse further samples. A thick smear from the sample was read for 30 minutes under oil-immersion microscope, the gametocytaemia was established at 1080gametocytes/µl considering that 1 µL of blood contains 8000 leucocytes (1.1% asexual parasitaemia). A 200 µL blood sample was extracted and the RNA extracted was diluted by 10, 100, 1000, 10 000; reverse transcription was performed on each of the dilutions and they were subsequently quantified by qPCR (Figs. 1 & 2).

In order to characterise and validate this technique the limit of detection of the technique was established using 5 separate gametocyte dilution series of field samples with precisely established gametocyte densities (thick blood smears examined on 1000 fields by two separate experienced microscopists). The limit of detection ranged from 80 to 160 gametocytes /mL (mean 119.26 ± 33.87). The reproducibility of the technique was also assessed by analysing the same extracted RNA 12 times in two different runs on two samples with different gametocyte densities (15.2 and 0.8 gametocyte/µL). The mean and standard deviation of these replicates were 11.23 gametocytes /µL (± 4.48) and 0.81 gametocytes /µL (± 0.34). A sample positive for *Plasmodium vivax* gametocytes was also tested, and confirmed that *P. vivax* gametocytes are not detected by this technique.

The method was also assessed on samples in EDTA-tubes from 49 patients prior to treatment at Day 0 of a malaria clinical episode in Madagascar. 1ml of blood was mixed with TRIzol® reagent and stored in liquid nitrogen until analysis. Of 49 patients analysed only 4.1% were positive with a thick smear whilst 20.4% were positive by quantitative RT-PCR (Table 1). The study was approved by the National Ethics Committee.


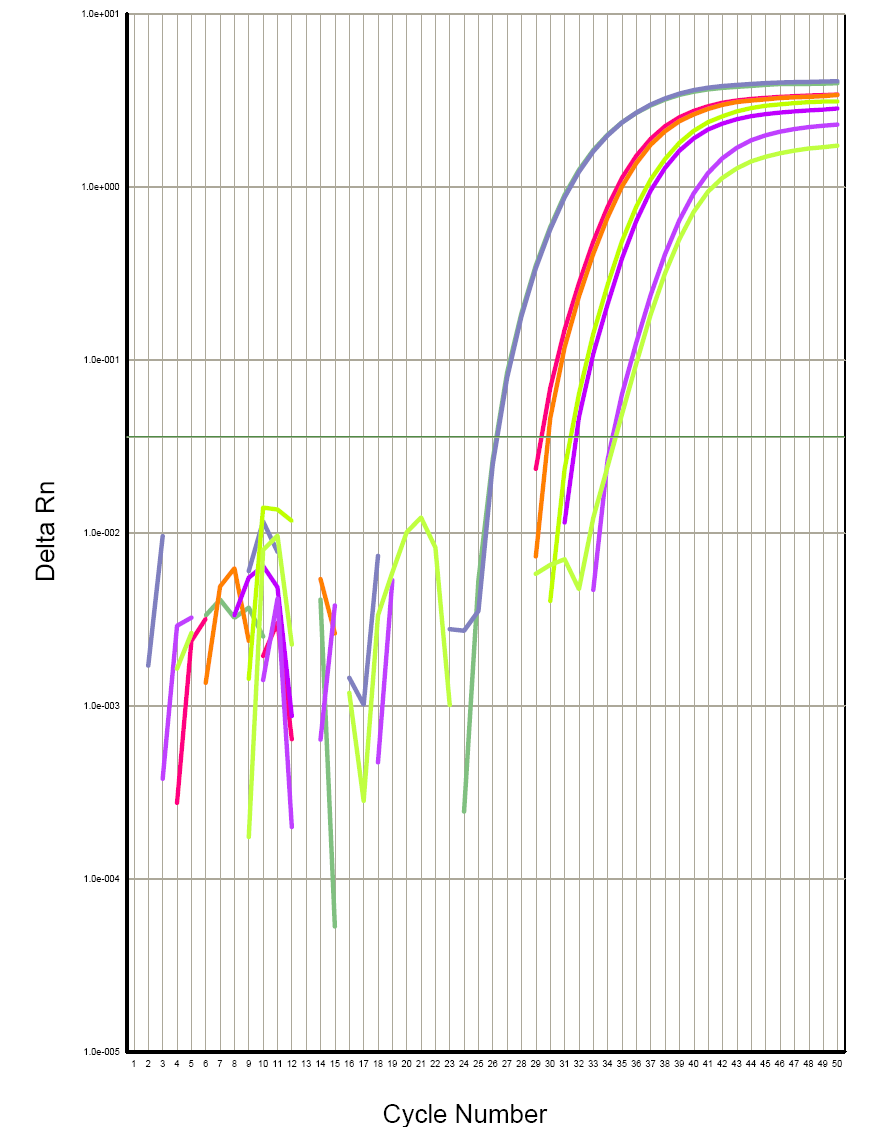


**Figure 1** Amplifications of dilutions of 1000, 100, 10, 1 gametocytes/µL from left to right (0.1 did not amplify). The cycle threshold (cycle at which significant fluorescence mirrors a relevant amplification) is directly correlated to the number of cDNA copies in a sample and establish that relationship to be Ct= -2.655092 Log(Conc) + 34.370285, shown in Figure 2.

**Figure 2.** Relationship between cycle threshold and log concentration of gametocytes.

**Table 1.** Assessment of RT-PCR gametocyte detection method in 49 samples at the time of clinical presentation compared with blood smear (200 microscopic fields read). The asexual parasite densities were: (i) 11.8 & 64.5 asexual parasites / μL in gametocyte positive by blood smear (N=2), (ii) 55.8 asexual parasites / μL ±SE 37.8, range 3.8-116.3 in gametocyte positive by RT-PCR but negative by blood smear (N=8) and (iii) 58.8 asexual parasites / μL ±SE 59.0, range 1.2-190.5 in gametocyte negative by RT-PCR and by blood smear (N=39).

|  |  | Blood smear | RT-PCR | | |
| --- | --- | --- | --- | --- | --- |
|  | N | Gametocyte density/μL | Number Gametocyte | Gametocyte density/μL | Number Gametocyte |
|  |  | Mean (range) | Positive | Mean±SE (range) | Negative |
|  |  |  |  |  |  |
| Gametocyte Positive | 2 | 16 (16&16) | 2 | 1.28 (0.9 & 1.65) | 0 |
| by blood smear |  |  |  |  |  |
|  |  |  |  |  |  |
| Gametocyte Negative | 47 | - | 8 | 0.6±0.66 (0.08-1.88) | 39 |
| by blood smear |  |  |  |  |  |
